# The CD31 molecule: a possible neuroprotective agent in acute ischemic stroke?

**DOI:** 10.1186/s12959-017-0134-4

**Published:** 2017-04-13

**Authors:** Tobias Boeckh-Behrens, Justus Kleine, Johannes Kaesmacher, Claus Zimmer, Lucas Schirmer, Sophie Simon, Holger Poppert

**Affiliations:** 1 Department of Neuroradiology, University Hospital Rechts der Isar, Technical University Munich, Ismaninger Str. 22, 81675 Munich, Germany; 2 Department of Neuroradiology, Vivantes Klinikum Neukölln, Rudowerstr. 48, 12351 Berlin, Germany; 3 Department of Neurology, University Hospital Rechts der Isar, Technical University Munich, Ismaninger Str. 22, 81675 Munich, Germany

**Keywords:** Stroke, Mechanical thrombectomy, Thrombus, Histology, CD31, Inflammation

## Abstract

**Background:**

The transmembrane receptor molecule CD31 is known to have immunomodulatory functions, suggesting a possible neuroprotective effect in the context of acute ischemic stroke by restricting an over-activation of secondary immunological processes. This study examines the density of CD31^+^ cells in mechanically extracted thrombi of stroke patients with the aim to test whether the occurrence of CD31^+^ cells was associated with a beneficial clinical outcome in those patients.

**Methods:**

Thrombi of 122 consecutive patients with large anterior circulation stroke were collected during intracranial mechanical recanalization. Out of these, 86 immunostained specimens of adequate quality could be analysed. The density of CD31^+^ cells was quantified and compared with clinical outcome data of the affected patients.

**Results:**

The density of CD31^+^ cells was positively related to early patient improvement (ΔNIHSS, *r* = 0.283, *p* = 0,012) with an even clearer relationship after exclusion of patients who died in the early hospital phase (*r* = 0.371, *p* = 0.001). This finding stayed stable also in the multivariate analysis after corrrection for other outcome-influencing factors (*p* = 0.049).

**Conclusion:**

This study shows a stable relation between CD31^+^ cells and early clinical improvement of patients with acute ischemic stroke. This finding is in line with recent reports showing immunomodulatory and potential neuroprotective effects of CD31, suggesting that CD31 may be a promising neuroprotective agent in stroke patients.

## Background

Inflammatory processes in the brain tissue after acute ischemic stroke are an important determinant of clinical course and patient outcome. Furthermore, increasing evidence highlights a strong linkage between thrombus formation and inflammatory pathways [1]. These assumptions are widely accepted, but with the results of the big recanalization studies being taken into clinical practice the opportunity is now open to recognizing the magnitude of the effect of these inflammatory processes with their inherent therapeutic possibilities.

The inflammatory cascade initiated by brain ischemia and tissue damage consists of highly complex, partially mutually reinforcing, partially counterdirectional events that are still not fully understood in terms of their interactions [2]. Although most post/peri-stroke inflammatory processes seem to have detrimental effects on patient outcome, certain neuroprotective or neuroregenerative properties are also apparent.

With the ability to obtain human in-vivo thrombi by mechanical extraction, it is now self-evident to address these questions by histopathological analysis. In previous work, we have found a relation between white blood cell count in stroke thrombi [3], as well as peripheral blood leukocyte count [4], and an unfavorable patient outcome, prompting the question of which leukocyte subpopulations are responsible for either beneficial or detrimental effects. One study adressing this question found no clear relation between clinical parameters and CD4^+^ T cells and CD68^+^ monocytes [5] in stroke thrombi. Another study found higher CD3^+^ T cells in clots from strokes of atherothrombotic origin compared to all other causes, without association to outcome parameters [6].

Recent reports suggest immunomodulatory effects of the cell adhesion molecule CD31 (PECAM-1) [7], supported by results that could show protective effects in the context of cardiovascular [8] and atherothrombotic [9] diseases. Here, we examined the presence and density of CD31^+^ cells in mechanically extracted thrombi of stroke patients. We aimed to test whether the occurrence of CD31^+^ cells was associated with a beneficial clinical outcome in those patients.

## Methods

Between 10/2010 and 09/2012, mechanically extracted thrombi of 122 patients with acute anterior circulation stroke were prospectively collected and processed as previously described [3] (all patients with anterior circulation stroke from our last published series [10]).

As primary endpoint, we investigated the relation between the density of CD31^+^ cells in the thrombus tissue and clinical outcome parameters.

HE-staining and semi-automated quantification of main thrombus components (fibrin/platelet aggregations [F/P], red blood cells [RBC], white blood cells [WBC]) of the paraffin-embedded thrombi were carried out as previously described [3]. CD31 immunostaining was done using an monoclonal mouse antibody, clone JC70A (Dako Deutschland GmbH, Hamburg, Germany), dilution 1:100. Primary antibody staining was carried out overnight at room temperature. Prior to primary antibody staining, incubation with 10% normal goat serum in 1x phosphate buffered saline (PBS) was performed. An affinity purified biotinylated goat anti-mouse IgG antibody (Vector Laboratories, Burlingame, CA) was used as antiserum followed by avidin-peroxidase (Vectastain ABC; Vector). Diaminobenzidine (Invitrogen, Carlsbad, CA) was used as chromogenic substrate. Negative control sections without primary antibodies were processed in parallel.

In a first step, all immunostained specimens were independently screened by two experienced investigators and assigned to a 3-point quality scale from 0 = insufficient through 1 = sufficient to 2 = good quality. All insufficient stainings were excluded, resulting in a total of 86 analyzable samples. Total numbers and distribution of CD31^+^ cells was quite heterogeneous between both different specimens and different regions of the same specimen. Hence, CD31^+^ cells were independently quantified by two investigators using a semiquantitative 5-point scale (0 = no cells, 1 = sporadic, 2 = few, 3 = some, 4 = many cells). The readers were blinded with regard to clinical and interventional data. In cases of discrepancies, results were reached by consensus.

As primary outcome parameter, the National Institute of Health Stroke Scale (NIHSS) was determined on admission and at discharge by the respective responsible neurologist, the modified Rankin Scale (mRS) up to 90 days post stroke was assessed by personal clinical examination, by phone interview, or by evaluating the medical reports of the rehabilitation centers.

Basic clinical and interventional data including age and sex, blood leukocyte count at admission and at day 1, occlusion site, time from symptom onset to reperfusion, number of retraction maneuvers, application of intravenous alteplase, stroke etiology according to the Trial of Org 10172 in Acute Stroke Treatment (TOAST) [11] classification, and recanalization success according to the thrombolysis in cerebral infarction (TICI) score [12] were assessed, as summarized in Table 1.

**Table 1:** Clinical patient characteristics

Characteristic	*n* = 86
Age (years): median, range	72, 21–92
Sex	
Female	40 (47%)
Occlusion site	
MCA	50 (58%)
ICA including carotid-T	22 (26%)
Combined ICA and MCA/ACA	13 (15%)
ACA	1 (1%)
Baseline NIHSS (range, median), *n* = 81 (94.2%)	15, 2–27
IV rtPA	57 (66%)
Time symptom onset to reperfusion (min): median, range, *n* = 70 (81.4%)	280 (0–495)
mRS (90 days), *n* = 41 (47.7%)	
0–2	17 (39.1%)
> 2	24 (60.9%)
Stroke etiology (TOAST), *n* = 85	
1 = arterioembolic	16 (18.8%)
2 = cardioembolic	40 (47.1%)
4 = other determined cause	9 (10.6%)
5 = cryptogenic	20 (23.5%)

Basic demographic and clinical patient characteristics (*MCA* middle cerebral artery, *ICA* internal carotid artery, *ACA* anterior cerebral artery, *IV rtPA* intravenous recombinant tissue plasminogen activator)

The two-sided Spearman’s rank correlation coefficient was used to assess correlations between the amount of CD31^+^ cells and primary clinical variables and outcome variables (NIHSS at admission and at discharge, ΔNIHSS between admission and discharge, mRS until 90 days). Differences in CD31^+^ densities were assessed by the nonparametric Kruskal–Wallis test, and boxplot diagrams were generated. For multivariate analysis, a logistic regression model was used to calculate the effect of several factors (age, recanalization success, NIHSS at admission, and time from symptom onset to reperfusion) on a dichotomized outcome parameter. Dichotomization was performed according to previous reports [13] using the following scheme: either a difference between baseline NIHSS and NIHSS at discharge ≥8 or NIHSS at discharge ≤1 as cut-off between major or minor neurological improvement. SPSS Statistics version 23.0 (SPSS Inc., IBM, Ehningen, Germany) was used for all statistical analyses.

## Results

### Demographic and clinical patient data

Most extracted thrombi (58%) were from isolated occlusions of the medial cerebral artery (MCA); the dominant stroke etiology was cardioembolic (47.1%); and most patients were heavily affected with a median NIHSS at admission of 15. Two-thirds of the patients received intravenous thrombolysis.

Baseline clinical and interventional data of all 86 patients with specimens of sufficient staining quality are summarized in Table 1.

### Correlation analyses

Quantitative analyses of main thrombus components showed median proportions of RBCs of 47% (3–96%), F/P of 44% (2–89%), and WBCs of 6% (1–25%).

The initial correlation analysis (Spearman-Rho, two-sided) showed no association between the amount of CD31^+^ cells and other histopathological parameters like RBC, F/P and WBC count inside the clot or leukocyte count in the peripheral blood. Additional group comparisons showed no significant differences between the CD31+ cell density groups concerning the amount of other clot components (RBC: p = 0.373, F/P: *p* = 0.276, WBC: *p* = 0.833)

There were also no correlations with the initial clinical appearance (NIHSS pre-treatment) or the clinical status at discharge (NIHSS post). However, a positive correlation with NIHSS improvement (ΔNIHSS) was apparent (r = 0.283, p = 0,012). To exclude possible coincidental or interfering effects, an additional multivariate logistic regression model was used that included age, recanalization success, received thrombolytic therapy, NIHSS at admission, and time from symptom onset to reperfusion as possible relevant outcome-influencing factors. Dichotomization of NIHSS improvement was done as described earlier.

In the multivariate analysis, the effect of CD31+ cells on NIHSS improvement was, although shortly not significant, still visible after correcting for the factors mentioned above (*p* = 0.057), as well as the effects of age (*p* = 0.051) and time to reperfusion (*p* = 0.002). Successful recanalization showed no effect on early patient improvement in the multivariate analysis, probably due to few cases with unsuccessful recanalization (TICI 0–2a, *p* = 0.999), as well as NIHSS at admission (*p* = 0.569) and received thrombolytic therapy (*p* = 0.497).

**Fig. 1:**
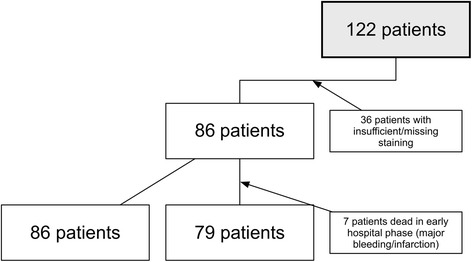
Study inclusion flowchart. Composition of the examined study population

In a second step, assuming that this possible positive effect may cannot manifest itself completely if the patient suffers from a major bleeding or malignant infarction, we performed an additional subgroup analysis with the purpose of further specifying the group of patients who may have the largest benefit of the described effect. Therefore, we excluded all patients who died in the early stroke phase up to discharge (*n* = 7), resulting in a patient subgroup of 79 patients, according to the scheme shown in Fig. 1.

As expected, the observed relation between the NIHSS improvement and the CD31^+^ count was even more evident in the subgroup correlation (*r* = 0.371, *p* = 0.001) and multivariate analysis (*p* = 0.049). The results of the correlation analysis of CD31^+^ cells and outcome parameters are summarized in Table 2.

**Table 2:** Correlation analysis

		CD31	NIHSS pre	NIHSS post	ΔNIHSS	mRS 90 days
CD31	Correlation coefficient (r) Significance (p) n	1.00	0.035	−0.151	**0.371**	−0.095
		—	0.767	0.201	**0.001**	0.593
		79	75	73	**72**	34

Correlation analysis of clinical outcome parameters and the amount of CD31^+^ cells. (Bold type of the ΔNIHSS values indicate significance)

**Fig. 2:**
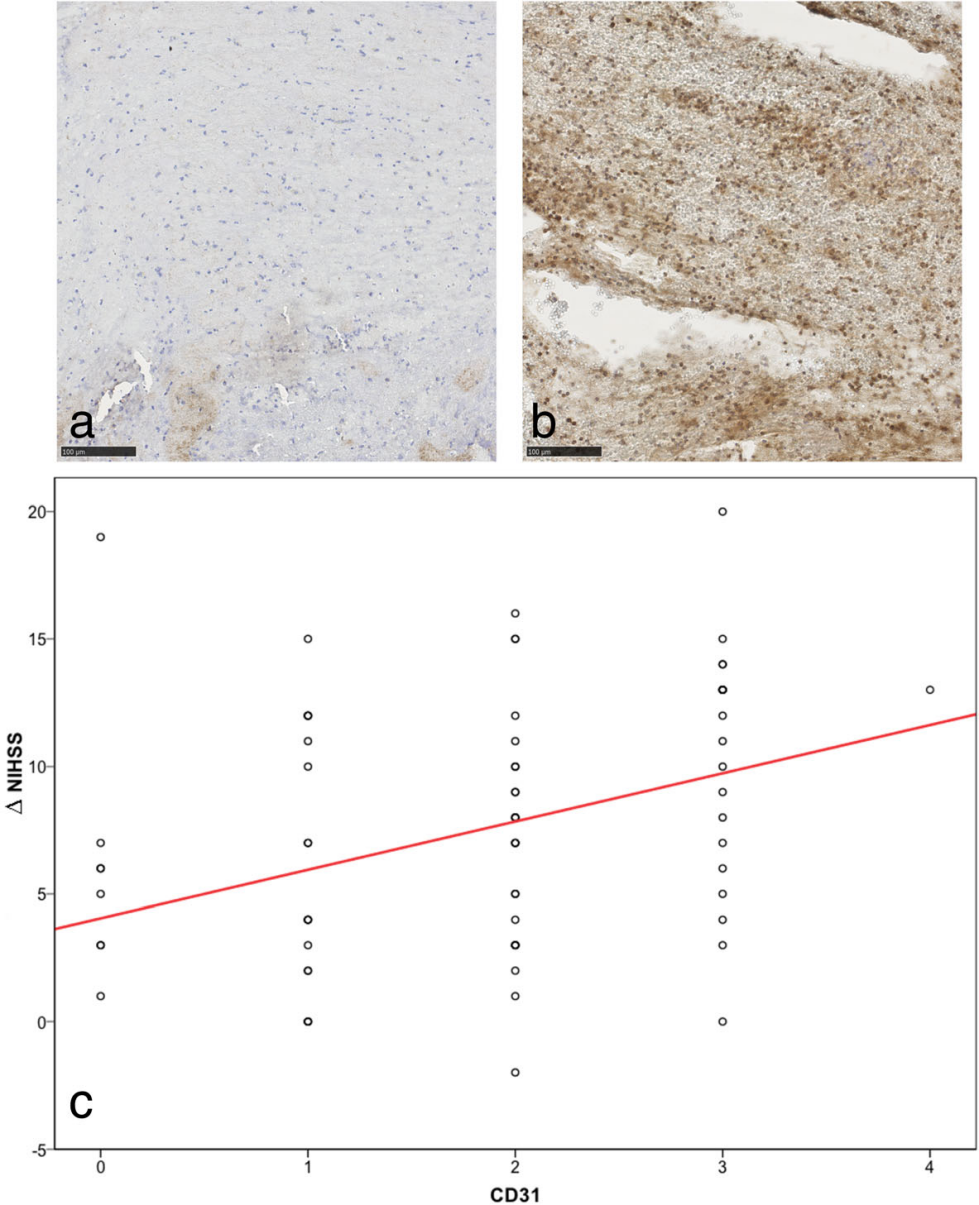
Relation between CD31^+^ cells and early patient improvement. Example of an immunostained specimen with almost no stained nucleated cells (**a**) and an example with many CD31^+^ stained cells (**b**). In this example, there is almost no "staining-negative" cell visible. **c** Boxplot analysis with regression line in red, showing the relationship between amount of CD31^+^-cells (0 = no cells, 1 = sporadic, 2 = few, 3 = some, 4 = many) and early patient improvement (represented by ΔNIHSS)

Figure 2 shows representative clot samples with low and high densitities of CD31^+^ cells as well as the boxplot analysis illustrating relationship and variance of the amount of CD31^+^ cells with regard to early clinical patient improvement (ΔNIHSS):

## Discussion

To our concern this study contains by far the largest number of patients with an immunohistochemical analysis of CD31^+^ cells in mechanically retrieved clots. We can show a clear relationship of the amount of CD31^+^ cells in extracted stroke thrombi with early clinical improvement. First, it seems surprising that one certain cell type inside the vessel-occluding thrombus material should have a measurable impact on patient outcome parameters. On the other hand, several published data as well as pathophysiological considerations support the plausibility of our results.

The CD31 molecule is a receptor protein expressed by multiple cell types involved in coagulatory and immunological processes (endothelial cells, platelets, and all types of leukocytes). Recent reports indicate a broad spectrum of different functions of this molecule [7], possibly affecting secondary processes in the course of acute stroke. Most importantly, CD31 seems to be able to modulate focal and excessive neuroinflammatory processes in the early post-stroke phase without affecting patients’ immunocompetence in general. This can be achieved by two main modes of action: Adhesional functions of CD31 might be required to re-establish endothelial integrity, which is compromised, for example, by hypoxic stress [7, 14]. In this context, Graesser et al. [15] showed that CD31 can protect against excessive neuroinflammation, presumably by limiting the migration of immunocompetent cells in the extravasal compartment. Additionally, and presumably more important, is the signaling function of CD31 leading to raised activation thresholds of lymphocytes (T and B cells) and subsequent decreased production of inflammatory mediators [16]. This downregulation of inflammatory pathways within ischemic brain lesions may protect stressed neurons against overactivated microglia [17] (by reducing their activation level). These well-known processes can lead to secondary infarct growth in the first few days after the initial stroke. Therefore, as CD31 very probably can inhibit these detrimental secondary effects in the early post-stroke phase, the observed positive effect on early patient improvement seems plausible from a pathophysiological point of view. Of course do these assumptions apply, if the amount of CD31 inside the thrombus material reflects its concentration in the peripheral blood which is supposable but has to be confirmed in further studies.

However, the observed early beneficial effects do not carry over to a better outcome after 3- months. From our initial patient cohort 3-month mRS values were available in only about half of these cases (34 patients), so perhaps the effect is simply masked due to the low patient number. Additionally, although the mRS value represents the standard outcome parameter in most stroke studies, it is known that this parameter is of limited accuracy compared with the NIHSS score, especially in older patients [18], probably also contributing to the loss of an observable long-term-effect.

A possible neuroprotective effect of CD31 in acute stroke is of special interest, as two studies showed the dependence of the immunmodulating effect of CD31 on the molecular integrity of the extracellular domains [19]. If this integrity is lost by enzymatic shedding, it seems to be possible to re-activate the immunmodulating signaling pathway by administering a CD31 derived peptide, which binds at the extracellular domain six of the truncated molecule [20]. This in turn implicates a possible neuroprotective treatment option in acute stroke patients, especially targeting the acute hyperinflammatory reaction in post-ischemic brain parenchyma in the early post-stroke phase. To follow-up, we plan to analyze CD31 counts (soluble parts and cell-bound molecules) in the peripheral blood of acute stroke patients prospectively. In addition, the neuroprotective effect of the CD31 based peptide will be evaluated in a mouse model of acute stroke.

### Limitations

Although our study contains the largest published series of immunohistochemically analyzed thrombi to date, the loss of nearly one-third of the initially included patients might implicate a potential selection bias. As the major cause for exclusion of the patients was low tissue quality (mostly due to technical staining difficulties, variable preservation times after retrieval, and in some cases not enough thrombus material), a systematic bias concerning the target endpoint seems extremely unlikely.

For quantification of the amount of CD31^+^ cells, we decided to use a semiquantitative 5-point-grading scale rather than manual or automated cell counting. This approach may be questioned as unusual, but, in our experience, exact quantification of immunostainings of thrombus specimens by automated or manual cell counting is a complicated and error-prone process: First, stroke thrombi are of very different sizes, leading to problems in including all thrombus fragments in the counted fields without including overlapping regions and doubled countings. Secondly, the immunostainings are often inhomogeneous in the staining intensity, between different specimens as well as on one slide. In our opinion, a grading system is most suitable to mitigate these kind of difficulties and to avoid a "pseudo-accuracy" that is not covered by the underlying data quality. Nevertheless, as complete standardization of immunhistochemical methods is not feasable, some misassignements to the specified groups are possible due to staining inhomogenities.

## Conclusion

This study shows a stable relation between CD31^+^ cells and early clinical improvement of patients with acute ischemic stroke. This finding is in line with recent reports showing immunomodulatory and potential neuroprotective effects of CD31, suggesting that CD31 may be a promising neuroprotective agent in stroke patients.

## Abbreviations

ACA: Anterior cerebral artery
F/P: Fibrin/platelet aggregations
ICA: Internal carotid artery
IV rtPA: Intravenous recombinant tissue Plasminogen Activator
MCA: Middle cerebral artery
mRS: Modified rankin scale
NIHSS: National Institute of Health Stroke Scale
RBC: Red blood cells
TICI: Thrombolysis in Cerebral Infarction
TOAST: Trial of Org 10172 in Acute Stroke Treatment
WBC: White Blood Cells
